# Characterization of immunogenicity of avian influenza antigens encapsulated in PLGA nanoparticles following mucosal and subcutaneous delivery in chickens

**DOI:** 10.1371/journal.pone.0206324

**Published:** 2018-11-01

**Authors:** Tamiru Negash Alkie, Alexander Yitbarek, Khaled Taha-Abdelaziz, Jake Astill, Shayan Sharif

**Affiliations:** 1 Department of Pathobiology, Ontario Veterinary College, University of Guelph, Guelph, ON, Canada; 2 Department of Biology, Wilfrid Laurier University, Waterloo, Canada; 3 Pathology Department, Faculty of Veterinary Medicine, Beni-Suef University, Al Shamlah, Beni-Suef, Egypt; Instituto Butantan, BRAZIL

## Abstract

Mucosal vaccine delivery systems have paramount importance for the induction of mucosal antibody responses. Two studies were conducted to evaluate immunogenicity of inactivated AIV antigens encapsulated in poly(D,L-lactide-co-glycolide) (PLGA) nanoparticles (NPs). In the first study, seven groups of specific pathogen free (SPF) layer-type chickens were immunized subcutaneously at 7-days of age with different vaccine formulations followed by booster vaccinations two weeks later. Immune responses were profiled by measuring antibody (Ab) responses in sera and lachrymal secretions of vaccinated chickens. The results indicated that inactivated AIV and CpG ODN co-encapsulated in PLGA NPs (2x NanoAI+CpG) produced higher amounts of hemagglutination inhibiting antibodies compared to a group vaccinated with non-adjuvanted AIV encapsulated in PLGA NPs (NanoAI). The tested adjuvanted NPs-based vaccine (2x NanoAI+CpG) resulted in higher IgG responses in the sera and lachrymal secretions at weeks 3, 4 and 5 post-vaccination when immunized subcutaneously. The incorporation of CpG ODN led to an increase in Ab-mediated responses and was found useful to be included both in the prime and booster vaccinations. In the second study, the ability of chitosan and mannan coated PLGA NPs that encapsulated AIV and CpG ODN was evaluated for inducing antibody responses when delivered via nasal and ocular routes in one-week-old SPF layer-type chickens. These PLGA NPs-based and surface modified formulations induced robust AIV-specific antibody responses in sera and lachrymal secretions. Chitosan coated PLGA NPs resulted in the production of large quantities of lachrymal IgA and IgG compared to mannan coated NPs, which also induced detectable amounts of IgA in addition to the induction of IgG in lachrymal secretions. In both mucosal and subcutaneous vaccination approaches, although NPs delivery enhanced Ab-mediated immunity, one booster vaccination was required to generate significant amount of Abs. These results highlight the potential of NPs-based AIV antigens for promoting the induction of both systemic and mucosal immune responses against respiratory pathogens.

## Introduction

Avian influenza viruses (AIV) are classified into low pathogenic and highly pathogenic viruses. Low pathogenic avian influenza (LPAI) viruses cause mild clinical signs and may affect egg production [1], whereas highly pathogenic avian influenza (HPAI) viruses cause massive influenza outbreaks and mortality in chickens [2]. However, various host and environmental factors may determine the pathogenicity of LPAI viruses [3]. In countries where both pathotypes circulate in poultry, whole inactivated and viral vectored vaccines are recommended to reduce the incidence and risks associated with AIV [4,5]. When administered parenterally, the systemic immunity induced by these vaccines provide partial to complete protection from disease progression, but generally does not prevent infection and virus shedding from infected birds [6,7]. This indicates the need to improve the immunogenicity and efficacy of existing AIV vaccines, which can be achieved by selecting adjuvants with superior ability to induce innate and adaptive immune responses [8,9], by exploring appropriate routes of vaccination [10] and by optimizing vaccine delivery methods [11–13].

CpG-ODN is one of the potent vaccine adjuvants identified for increasing the efficacy of many vaccines including AIV vaccines [14,15]. By interacting with Toll-like receptor (TLR) 21 in chickens [16] and TLR9 in mammals [17], CpG ODN triggers innate signaling pathways, which lead to cytokine and chemokine induction, which in turn, orchestrate adaptive immunity [18]. In addition to innate immune system cells, cells of the adaptive immune system are also activated by CpG ODN [19]. Previously, we have also shown that encapsulation of CpG ODN in biodegradable poly(D,L-lactide-co-glycolide) (PLGA) nanoparticles (NPs) enhances and sustains its adjuvant property and promotes high avidity antibody production when delivered with inactivated AIV [11].

AIV vaccines triggering mucosal immune responses along the intestine and the respiratory tract are ideal for preventing AIV transmission cycle by blocking virus replication at the primary sites of infection [20]. Mucosal vaccination (via oral, ocular and respiratory routes) is not an efficient way for delivery of non-replicating and subunit vaccines because of impaired vaccine uptake by immune cells due to various physiological barriers. Mostly, vaccines administered to mucosal surfaces are captured by the mucus for clearance by epithelial barriers and by proteolytic degradation [21,22] before interacting with the mucosal-associated lymphoid tissues [23,24].

Much has been learned from nanotechnology assisted mucosal vaccine delivery for inducing mucosal and systemic immune responses. These methods have advantages of prolonging antigen presentation, antigen dose sparing effects and protecting antigens from proteolytic degradation by mucosal enzymes [25,26]. Poly(D,L-lactide-co-glycolide) (PLGA), a biodegradable polymer approved for delivery of pharmaceuticals in humans has been widely used for the production of micro- and nano-particles (NPs) for entrapping or adsorbing vaccines [13]. Delivering PLGA NPs-based vaccines through the oral and nasal routes improved the immunogenicity of several recombinant and conventional vaccines derived from human and veterinary pathogens [27–29]. PLGA NPs are flexible and tunable in that their outer surface can be modified with other polymers such as chitosan or poly(β-amino esters) for more effective mucosal vaccine delivery [30,31]. The mucoadhesive property of chitosan and its derivative N-trimethyl chitosan allows better interactions of nanoparticles with mucus, which then improves the residence time of vaccines on mucosal surfaces and, thus, over time facilitates cellular uptake of antigens [32,33]. Furthermore, recent studies have shown that chitosan can act as a strong innate response inducer by activation of stimulator of interferon gene (STING) pathways [34], which have significant implications for orchestrating antigen-specific adaptive immune responses and as standalone antiviral agent [35]. Additionally, for the induction of potent and long lasting immune responses, molecules which can target mucosal antigen presenting cells (APCs) can be covalently attached to PLGA NPs [36]. In mouse experiments, mannan coated PLGA NPs improved the immunogenicity of mucosal vaccines [37]. Mannan may bind to mannose receptors on APCs or Microfold cells (M cells) residing at the inductive sites of the respiratory or intestinal tracts [24]. In the current study, we hypothesized that combined nasal and ocular administration of inactivated AIV antigens encapsulated in PLGA NPs with surface modifications confers mucosal and systemic antibody responses in specific pathogen free chickens in a prime-boost vaccination strategy.

## Materials and methods

### Animal experiments

Two experiments were conducted in this study. In the first experiment, in which subcutaneous vaccination was used, seven-days-old specific pathogen free (SPF) layer-type chickens (CFIA, Ottawa, Canada) were assigned to 7 groups (n = 6/group). Experimental groups and their description are presented in Table 1. The vaccine formulations used in this study consisted of 20 μg of PLGA encapsulated AIV and 1.8–2.1 μg CpG ODN per dose in 100 μL PBS. The doses for AIV and CpG ODN were selected based on our previous work [15,38,39]. Primary vaccination was done at 7 days of age, followed by the secondary vaccination at 21 days of age. Both primary and secondary vaccinations were administered subcutaneously in the neck region. Blood and lachrymal secretions were collected at weeks 1, 2, 3, 4 and 5 post-primary vaccination for antibody detection.

**Table 1:** Study design showing experimental groups and vaccination protocols.

Experiment 1 (Subcutaneous vaccination)			Experiment 2 (Mucosal vaccination)		
Groups	Prime vaccines	Booster vaccines	Groups	Prime vaccines	Booster vaccines
1	NanoAI	NanoAI	1	NanoAI+CpG	NanoAI+CpG
2	NanoAI	NanoAI+CpG	2	Mannan-NanoAI	Mannan-NanoAI
3	NanoAI+CpG	No booster	3	Chitosan-NanoAI	Chitosan-NanoAI
4	NanoAI+CpG	NanoAI	4	Inactivated AIV	Inactivated AIV
5	NanoAI+CpG	NanoAI+CpG	5	Mock Nano	Mock Nano
6	Virosomes	Virosomes			
7	Mock Nano	Mock Nano			

NanoAI = PLGA NPs encapsulating AIV alone; NanoAI+CpG = PLGA NPs co-encapsulating AIV and CpG ODN; Virosome = AIV-virosome; Mock Nano = non-encapsulating PLGA NPs; Mannan-NanoAI = PLGA NPs co-encapsulating AIV and CpG ODN and surfaced modified with mannan; Chitosan-NanoAI = PLGA NPs co-encapsulating AIV and CpG ODN surfaced modified with chitosan; Whole-virus inactivated AIV vaccine formulated with squalene-based oil-in-water adjuvant called AddaVax (InvivoGen, San Diego, CA, USA) is referred as Inactivated AIV. Virosomes were used as a control for subcutaneous route of vaccination and to produce antisera.

In the second experiment, in which mucosal vaccination was used, seven-days-old SPF layer type chickens (CFIA, Ottawa, Canada) were assigned to 5 groups (n = 8/group). Vaccination schemes and doses were the same as the first experiment. However, in this experiment, all vaccine doses were administered via the ocular and nasal routes (Table 1).

In all experiments, chickens were maintained in the animal isolation facility of the Ontario Veterinary College, University of Guelph. Animal experiments were approved by the University of Guelph Animal Care Committee. Accordingly, chickens were kept in groups in enriched isolators and supplied with feed and water *ad libitum*. At the end of the experiments, chickens were euthanized humanely using carbon dioxide inhalation.

### Avian influenza virus inactivation

The formalin inactivated whole avian influenza virus antigens were prepared as indicated in a previous study. At first, H4N6 (A/Duck/Czech/56) was propagated to passage four and five in 10-day-old embryonated SPF eggs via the allantoic route to obtain large quantities of allantoic fluid. Typically, allantoic fluid harvested from embryonated chicken eggs 72 hr post-inoculation with 4 hemagglutination units (HAU) of H4N6 attained a 50% tissue culture infective dose (TCID_50_)/mL of 2.87x10^7^ in MDCK cells. This allantoic fluid containing H4N6 harvested from embryonated SPF eggs 72 hr post-infection was diluted with HNE buffer and inactivated by formalin (final concentration 0.02%) for 72 hr at 37°C and was subjected to ultracentrifugation at a speed of 90,000 × g for 2 hr in 30% sucrose cushion using a SW32 Ti rotor (Optima L-80 XP—Beckman Coulter, Inc.) [40]. The pellet was dissolved with HNE buffer (pH 7.4) and further ultracentrifuged in sucrose gradients (10%/60%) at 154,000 × g for 4 hr in a SW32 Ti rotor. The hemagglutination activity and protein concentration were determined by hemagglutination and BCA (Thermo Scientific, Rockford, IL) assays, respectively. Formalin inactivated antigenic preparation from the last ultracentrifugation step was ten-fold serially diluted and inoculated into 10-day-old embryonated SPF eggs and incubated for 72 hr. Three consecutive passages were further conducted in the same way as in above. The presence or absence of infectious particles in allantoic fluid collected from all passages were evaluated by determining HA activity and TCID_50_ in MDCK cells [15].

### Preparation and surface modifications of PLGA NPs

AIV and CpG ODN-loaded PLGA NPs were prepared as described previously [11,41]. Briefly, CpG ODN (class B CpG ODN 2007, phosphorothioate backbone modified, 5'-TCGTCGTTGTCGTTTTGTCGTT-3') and polyethylenimine (branched, 25 kD) complex was made as described [42]. This complex (250 μL) and inactivated AIV (1250 μg /250 μL) were sonicated in 2500 μL of 4.5% PLGA solution. PLGA (Resomer® RG 503H, free carboxylic acid, 24–38 kD) was dissolved in dichloromethane. The resulting solution was sonicated in 6.25 mL of 2% polyvinyl alcohol. Similarly, PLGA NPs encapsulating inactivated AIV only (without CpG ODN) and mock PLGA NPs (non-encapsulating) were produced [43]. The emulsions were stirred for 4 hr for dichloromethane to evaporate. The PLGA NPs were pelleted at 20,000 × g for 30 minutes at 4°C, washed 3 times in DNase/RNase free water, resuspended, snap frozen and lyophilized.

The surface of PLGA NPs encapsulating AIV and CpG ODN were coated with mannan by carbodiimide conjugation [36,44]. Briefly, non-lyophilized PLGA NPs pellet (5 mg/mL) was dissolved in 2-(*N*-morpholino)ethanesulfonic acid buffer (MES buffer; 0.11 M; pH 5.2) and treated with 765 μg 1-ethyl-3-[3-dimethylaminopropyl]carbodiimide hydrochloride (EDC) and 2.295 mg N-hydroxysulfosuccinimide (s-NHS) (for each 10 mL containing 50 mg PLGA NPs) for 30 minutes at room temperature with mild agitation for activating the carboxylic acid on PLGA NPs. After washing with 1x cold PBS to remove the activating molecules and byproducts such as urea, the pellet was resuspended in 10 mL MES buffer and 100 mg mannan (derived from Saccharomyces cerevisiae) was added, and further incubated for 2 hr at room temperature with gentle stirring. Finally, the preparation was washed 3 times in water and lyophilized. Chitosan coated PLGA NPs encapsulating AIV and CpG ODN were produced as indicated [45]. Non-lyophilized PLGA NPs (100 mg), were resuspended in 5 mL of 0.5% chitosan solution (medium molecular weight chitosan, 75–85% deacetylated and dissolved in 1% glacial acetic acid, pH 5) and stirred for 4 hr to allow surface deposition of chitosan onto the PLGA NPs. The dispersion was washed 3 times and lyophilized for use. All major reagents and chemicals were from Sigma-Aldrich.

### Characterization of PLGA NPs

The size and surface charge of PLGA NPs, and encapsulation efficiency of CpG ODN were determined as described in our previous work [41]. The encapsulation efficiency of AIV antigen was determined as described in a previous work [46].

### In vitro release assay

A three-week *in vitro* release of AIV from the PLGA NPs was determined as previously described [47]. Briefly, 20 mg/mL PLGA NPs encapsulating AIV was re-suspended in PBS (containing 0.01% sodium azide salt) and incubated at 37°C with constant shaking. At predetermined time intervals, the suspension was centrifuged at 20,000 × g for 30 minutes. The supernatant was collected and the pellet was resuspended in 1 mL PBS and further incubated. The amount of protein released into the supernatant was determined using the BCA assay. Moreover, in this study, ELISA plates were coated with the supernatants collected during the release assay (similar amount of heat killed AIV also used to coat plates for comparison) to evaluate if the entrapped AIV antigen reacts with antibodies from chickens vaccinated with virosomes derived from the same AIV strain.

### In vitro phagocytosis of PLGA NPs

Phagocytosis of PLGA NPs was assessed using chicken bone marrow derived dendritic cells (BM-DCs). The BM-DCs were generated from mononuclear cells isolated from the femur of 3 weeks old SPF chickens as previously described [48], however, in our protocol we used recombinant chicken GM-CSF (50 ng/mL) and IL-4 (10 ng/mL) (Kingfisher Biotech, Inc. Saint Paul, MN) for mononuclear cells differentiation. The uptake of mannan coated PLGA NPs encapsulating rhodamine was evaluated by immunofluorescence microscopy.

### Virosome preparation and characterization

A virosome-based AIV vaccine was prepared as described previously [39]. Briefly, purified AIV (5 mg/mL) obtained by sucrose gradient ultracentrfigugation as indicated in previous section was UV-irradiated and virus envelope was solubilized with Octaethylene glycol (C_12_E_8_, pH 7.3), which was then followed by membrane reconstitution using methanol activated resin Bio-beads SM-2 (Bio-Rad laboratories) for virosome production [49,50]. Protein compositions of virosomes were determined by SDS-PAGE (Thermo Scientific) and Coomassie Brilliant Blue (Thermo Scientific) staining. The morphology of both viruses and virosomes was characterized and examined in FEI Tecnai G2 F20 transmission electron microscopy. The mean particle size distribution and zeta potential were determined by dynamic light scattering with a Malvern Nano ZS (Malvern Instruments). CpG ODN-polyethylenimine complex was added before envelope reconstitution to enhance CpG ODN encapsulation. Virosomes were solubilized with IGEPAL CA-630 (Octylphenoxy poly(ethyleneoxy)ethanol) (Sigma Aldrich) to release entrapped CpG ODN for quantification of CpG ODN encapsulation as described previously [41].

### Hemagglutination inhibition

Briefly, 4 HA units of H4N6 virus were added to two-fold serially diluted serum samples and incubated for 30 minutes. Subsequently, 0.5% chicken red blood cells were added and the results were read after 30 minutes. The hemagglutination inhibition (HI) titres were determined as the reciprocal of the samples resulting in complete inhibition of hemagglutination of chicken red blood cells [15].

### Enzyme-linked immunosorbent assay

AIV antigen-specific IgG, IgM and IgA in serum samples and lachrymal secretions were determined as previously described, with some modifications [11,15]. Briefly, 96-well polystyrene plates (Nunc, Maxisorp) were coated (125 ng/well) with virosomes prepared from H4N6. After blocking with PBS containing Tween-20 and 25% fish gelatin, diluted sera and lachrymal secretions were added and plates were incubated for 1 hr. Each sera and lachrymal secretions were assayed in duplicate. Horseradish peroxidase (HRP)-conjugated goat anti-chicken IgG or HRP-goat anti-chicken IgM (Bethyl Laboratories, Montgomery, Texas) was added for detecting IgG or IgM, respectively. Then, ABTS peroxidase substrate (Kirkegaard and Perry Laboratories Gaithersburg, Maryland, USA) was added and plates were further incubated. The reaction was stopped with 1% SDS and the plates were read with an ELISA plate reader at 405 nm (Bio-Tek Instruments, Winooski, Vermont USA).

For IgA determination in the lachrymal secretions, mouse anti-chicken IgA (AbD Serotec, Kidlington, Oxford, UK) was used as a primary antibody and HRP-goat-anti-mouse IgG (H/L) (AbD Serotec; Kidlington, Oxford, UK) as detecting antibody. Otherwise, all steps remain similar to IgG detections. All incubations were done at room temperature.

### Interferon (IFN)-γ assay

At day 35 post-primary vaccination (experiment 1), spleen mononuclear cells were isolated using Histopaque density gradient and were seeded into 48-well plates at a cell density of 2×10^5^ cells/mL in complete RPMI-1640 medium (Invitrogen, Burlington, ON). The cells (in triplicates) were stimulated for 72 hr with heat killed H4N6 (100 ng/well) and IFN-γ concentration in cell supernatants was determined using the chicken IFN-γ CytoSet kit (Invitrogen, CA, USA) according to the manufacturer’s instructions.

### Statistics

The data obtained from ELISA assays in all *in vivo* experiments were analyzed using general linear models of SAS with chicken as the experimental unit (SAS 188 9.3, Cary, NC) and comparisons were made within a defined single time points. Duncan’s Multiple Range test was used when a significant difference was observed among the groups. Kruskal-Wallis (a nonparametric) test was used to analyze data on *in vitro* assays and on hemagglutination inhibition (HI) titers. A value of *P*<0.05 was considered significant.

## Results

### PLGA NPs and virosome characterization

Previously, PLGA NPs encapsulating various classes of TLR ligands and AIV antigens were generated and their effects on innate immune system cells and efficacy for vaccine delivery were assessed [11,41]. The encapsulation efficiency of inactivated AIV in PLGA NPs ranged from 62–67%. To improve the efficacy of PLGA NPs as a mucosal vaccine delivery system, two types of surface modifications were applied to these nanoparticles. In one of the preparations, the surface of nanoparticles was coated with chitosan. Dynamic light scattering analysis indicated that chitosan coated nanoparticles had a net positive surface charge of +31 mV and a size of about 819 nm in diameter. Non-surface modified nanoparticles were relatively smaller in size and showed net negative surface charge (-36 mV). The size of mannan coated nanoparticles was about 719 nm in diameter with a net negative surface charge (-30 mV) and were found efficiently phagocytosed by DCs (data not shown).

**Fig 1:**
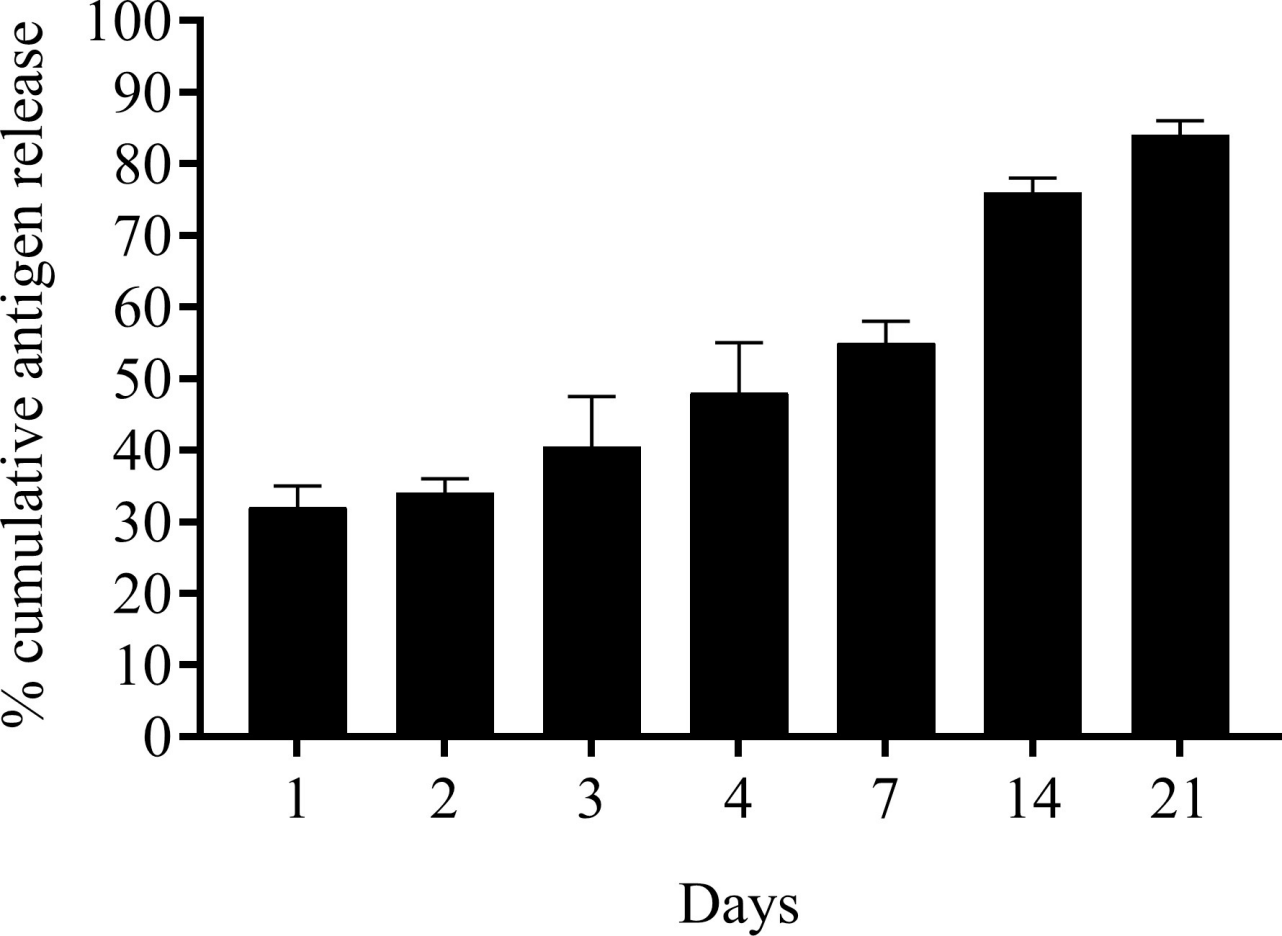
*In vitro* antigen release profile. A predetermined quantity of PLGA NPs encapsulated AIV in PBS (pH 7.4) was incubated at 37°C with constant shaking. The supernatants were collected at different time points and the amount of antigen (AIV) released from the NPs was assayed with BCA. The assay was conducted twice and results were presented as mean ± SEM.

The release of AIV antigen from the PLGA NPs showed a characteristic burst release in the first 24 hr followed by a sustained release over a three-week period when incubated at 37°C with agitation (Fig 1). We observed cumulative burst release of about 35% of encapsulated antigens within 24 hr and by week 3 over 85% of the encapsulated antigens were released. Moreover, the released AIV antigens displayed hemagglutinating activity and reacted in an ELISA assay with positive serum samples obtained from chickens vaccinated with virosomes.

The virosomes prepared for this study were further characterized by transmission electron microscopy. Lipid vesicles with spikes protruding from their membranes were commonly observed. Spike density as demonstrated by the presence of protruding electron dense materials from the native virus envelope was greater compared to spike density on vesicles (data not shown). Previous studies also revealed the incorporation of about 40% of the initial viral membrane proteins and approximately 50% of the initial viral lipids in the newly formed virosomes [51]. The diameter of the virosomes and the spherical virus was very similar, 89 nm for the virus and 90 nm for the virosomes. Virions with filamentous shapes and occasionally irregular morphology were also detected as in previous works [52]. Importantly, the virosomes maintained HA structural integrity.

### Antibodies generated in chickens by subcutaneously administered PLGA NPs

**Fig 2:**
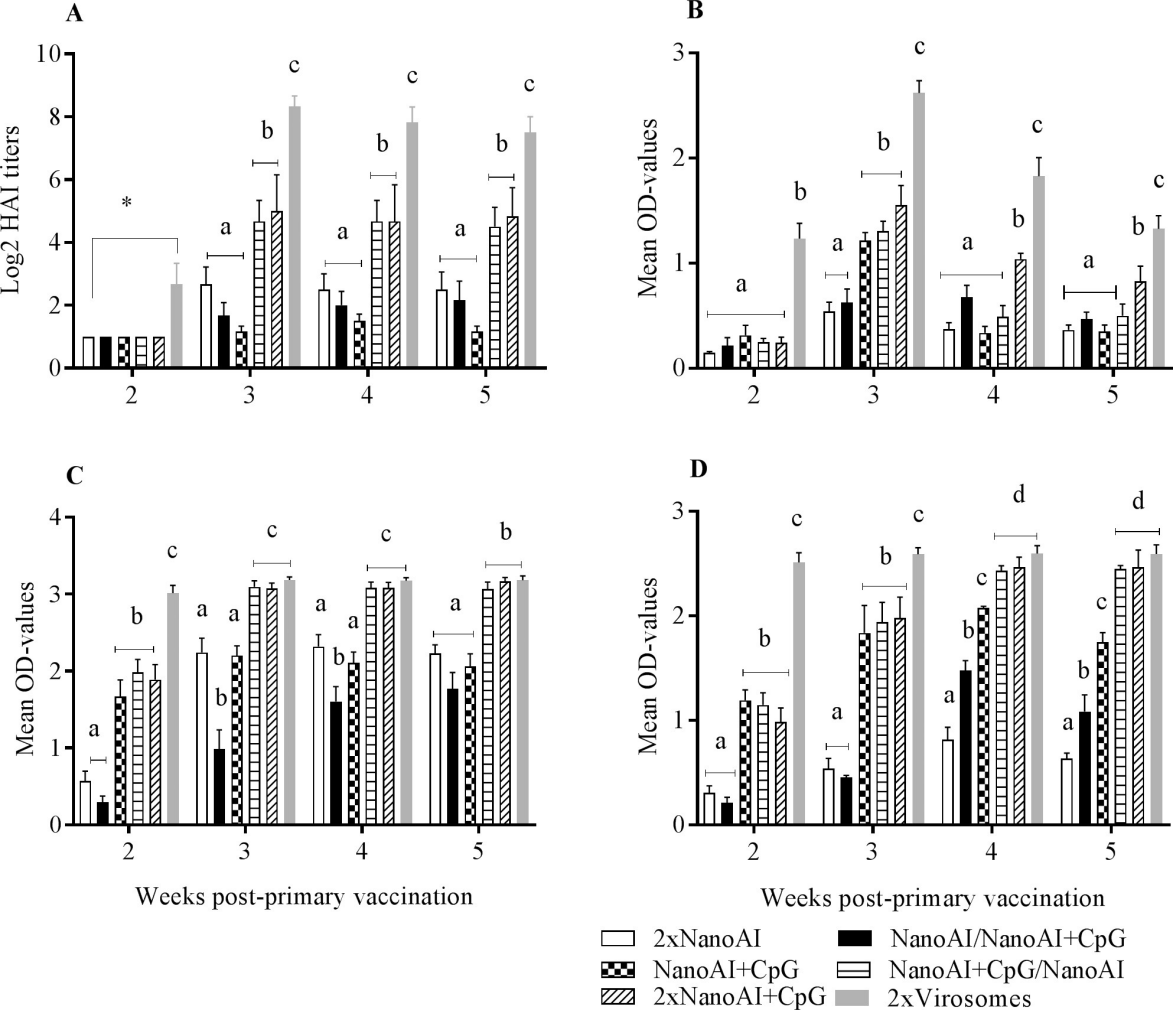
**Serum HI (A), serum IgM (B) and IgG (C), and lachrymal IgG responses (D)**. SPF chickens were vaccinated subcutaneously with PLGA NPs formulated AIV vaccines and virosomes. Serum samples and lachrymal secretions were collected weekly for antibody determination. Data shown were mean ± SEM, (n = 6/group). *P*<0.05 was considered significant. Different letters within a defined single-time point show significant differences between groups, while identical letters within a defined single time point show no statistically significant differences between groups. The cut-off value for ELISA was set as the mean OD value of the Mock Nano sera plus 2 standard deviations (SD) to ensure that 95% of the OD-values for the Mock Nano sera sample fell within this range.

Chickens immunized subcutaneously with AIV and CpG-ODN co-encapsulated in PLGA NPs (referred as 2x NanoAI+CpG) produced relatively higher HI titers (≥1:32) compared to a group immunized with AIV encapsulated in PLGA NPs (referred as 2x NanoAI), a group vaccinated with NanoAI and boosted with NanoAI+CpG (NanoAI/NanoAI+CpG) and with another group that received only the priming dose with NanoAI+CpG at weeks 3, 4 and 5 post-primary vaccination (p<0.05) (Fig 2A). Moreover, a vaccination scheme with NanoAI+CpG prime and NanoAI boost (NanoAI+CpG/NanoAI) resulted in HI titers (≥1:32) comparable to 2x NanoAI+CpG. Chickens immunized with a vaccine formulation that lacked the adjuvant component (2xNanoAI) had very low HI titer (≤1:8) similar to the group that received only a priming dose (NanoAI+CpG). The 2x virosomes (primed and boosted with virosomes) induced the highest HI titers at all time points (p<0.05). At week 1 post-primary vaccination, detectable HI titers were not observed in all groups.

To further test the immunogenicity of encapsulated AIV, AIV-specific IgM and IgG were determined in serum samples and lachrymal secretions. The results revealed that the inclusion of CpG ODN in the vaccine formulation, particularly 2x NanoAI+CpG and NanoAI+CpG/NanoAI induced higher serum IgM (OD-values of 1.8 and 1.4, respectively) at week 3 post-primary vaccination compared to formulations lacking CpG ODN such as 2x NanoAI or a group primed with NanoAI and boosted with NanoAI+CpG (NanoAI/NanoAI+CpG) (Fig 2B) (p<0.05) indicating the importance of including CpG ODN both in the prime and boost or at least in the priming dose for inducing a higher Ab response. The latter two groups had the lowest serum IgM at all time points. IgM responses decreased for all vaccines by weeks 4 and 5 post-primary vaccinations. As expected, the positive control, 2x virosomes, induced the highest serum IgM responses at all time points investigated.

By 2 weeks post-primary vaccination, 2x NanoAI+CpG, NanoAI+CpG and NanoAI+CpG/ NanoAI resulted in higher IgG in serum (Fig 2C) and lachrymal secretions (Fig 2D) compared to those two groups vaccinated with AIV encapsulated in PLGA NPs, but lacking the adjuvant component (p<0.05). The 2x NanoAI and NanoAI/NanoAI+CpG had the lowest IgG in both serum and lachrymal secretion at multiple time points investigated indicating the absence of CpG ODN in both prime and boost or its absence in the prime dose impaired Ab responses. Compared to all other nanoparticle groups, 2xNanoAI+CpG, and NanoAI+CpG primed and NanoAI boosted groups (NanoAI+CpG/NanoAI) mounted higher serum IgG (OD values of 3.1) as well as higher lachrymal IgG (OD-values of 2.5), particularly at weeks 4 and 5 post-primary vaccination (p<0.05). At these latter time points, IgG responses in these two groups approached that of virosome group. All vaccines (nanoparticle-based and virosomes) delivered through the subcutaneous route did not elicit detectable amounts of IgA in serum samples and lachrymal secretions of all vaccinated groups.

### In vitro stimulation of splenocytes

**Fig 3:**
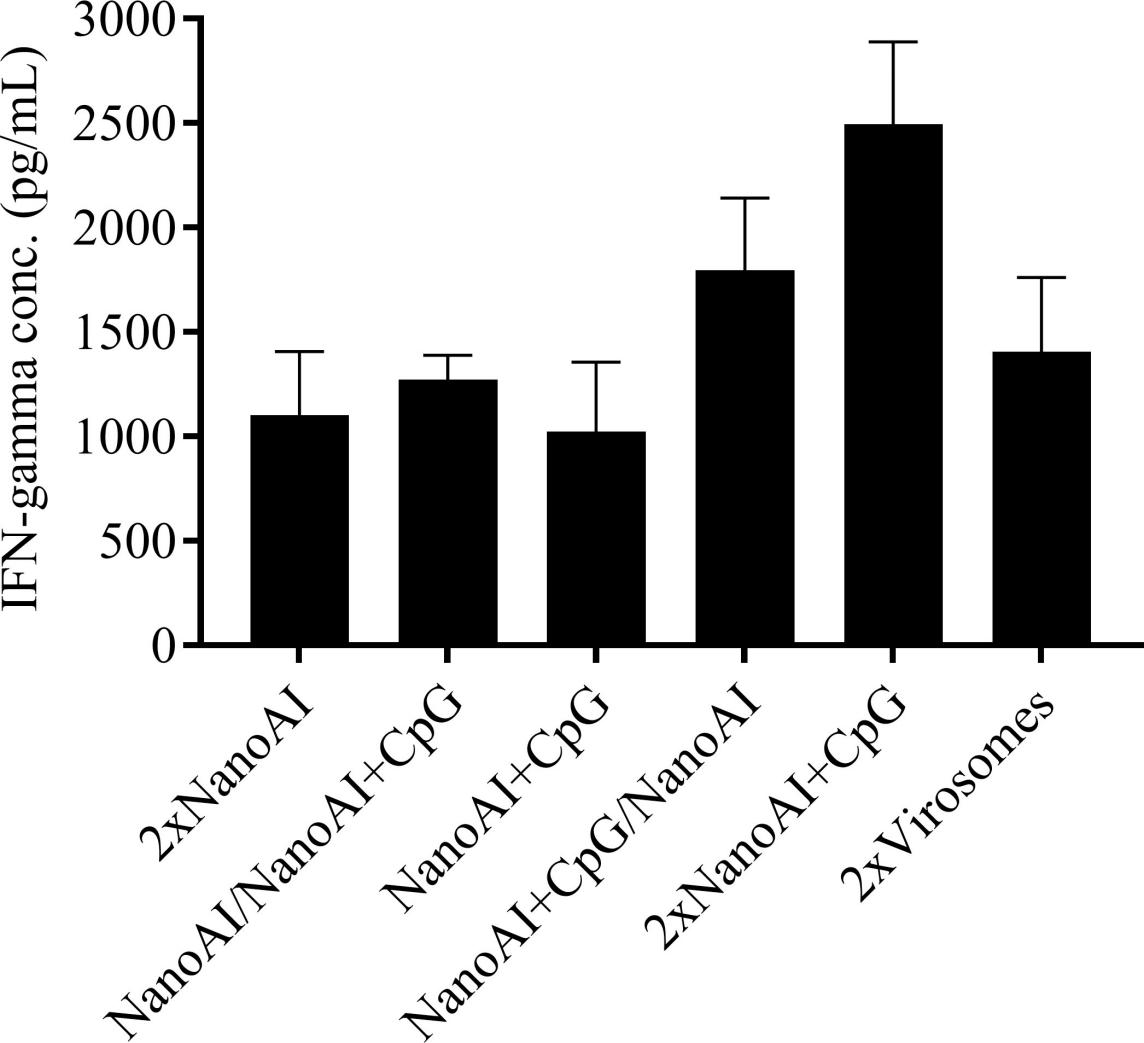
Evaluation of IFN-γ production by *in vitro* stimulated splenocytes with heat killed AIV. Splenocytes were harvested on day 35 post-primary vaccination from SPF chickens immunized subcutaneously with PLGA NPs based AIV vaccines and virosomes. They were stimulated with heat killed AIV (100 ng/well) for 72 hr and IFN-γ was measured in the cell supernatants. Results represent mean ± SEM. *P*<0.05 was considered significant.

Although not significantly different from other vaccine formulations, splenocytes from 2xNanoAI+CpG immunized chickens produced larger quantities of IFN-γ upon re-stimulation with heat killed AIV (Fig 3).

### Antibodies generated in chickens by mucosally delivered PLGA NPs

**Fig 4:**
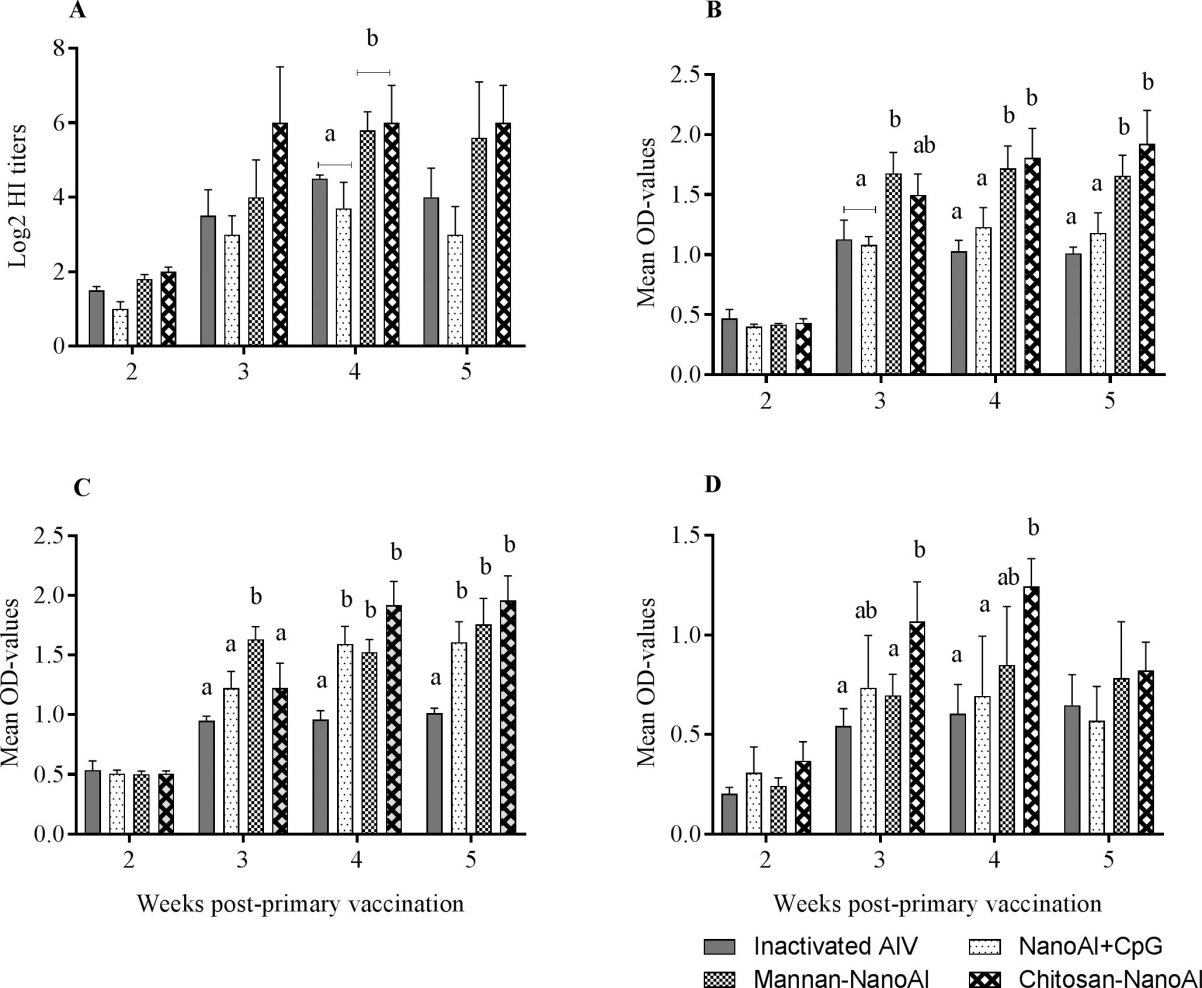
**Serum HI titers (A) and serum IgG (B), and lachrymal IgG (C) and IgA (D)** induced by surface modified PLGA NPs formulated AIV vaccines after nasal and ocular applications. One-week-old SPF chickens were vaccinated mucosally with nanoparticle formulated AIV vaccines or with whole-virus inactivated vaccine. Serum samples and lachrymal secretions were collected weekly for antibody determination. Data shown were mean ± SEM (n = 8/group). *P*<0.05 was considered significant. Different letters within a defined single time point show significant differences between groups, while identical letters within a defined single time point show no statistically significant differences between groups.

Mucosal application of surface modified PLGA NPs induced higher amounts of antibody responses, constituting both systemic and mucosal responses. At week 4 post-primary vaccination, both chitosan coated PLGA NPs encapsulating AIV and CpG ODN (Chitosan-NanoAI) and mannan coated PLGA NPs (Mannan-NanoAI) induced higher HI titers compared to PLGA NPs encapsulating AIV and CpG-ODN (NanoAI+CpG) and the vaccine containing inactivated whole-virus (Fig 4A) (p<0.05). Chitosan-NanoAI and Mannan-NanoAI induced large amounts of HI (≥1:64) at weeks 3 and 5 post-primary vaccination.

Immunogenicity of surface modified PLGA NPs encapsulated AIV and CpG ODN was further assessed by evaluating mucosal and systemic IgG and IgA responses. Chitosan-NanoAI and Mannan-NanoAI, administered via combined nasal and ocular routes induced higher amounts of serum IgG (OD-values of 1.6) from week 3 post-primary vaccination compared to the other two groups (Fig 4B). At week 2 post-primary vaccination, each of the four vaccine formulations did not induce significant amounts of serum as well as lachrymal antibodies. Regarding lachrymal IgG production, the three vaccine formulations namely, NanoAI+CpG, Mannan-NanoAI and Chitosan-NanoAI induced higher responses starting by week 3 post-primary vaccination (p<0.05). These groups induced significantly higher lachrymal IgG (OD-values ranging from 1.5–2.2) by week 4 and 5 post-primary vaccinations compared to inactivated AIV formulated with squalene-based oil-in-water adjuvant, also called AddaVax (Fig 4C). Mucosal delivery of PLGA NPs decorated with chitosan induce large quantities of IgA (OD-values ranging from 1.25–1.4) at weeks 3 and 4 post-primary vaccinations compared to the rest of the groups; still the other polymer based particulate vaccines induced detectable IgA at later time points (Fig 4D).

## Discussion

Recent studies in pigs and chickens have shown an increase in the immunogenicity of inactivated influenza vaccines encapsulated in PLGA NPs or chitosan NPs [11,43,53]. In the present study, we showed that inactivated AIV and CpG ODN encapsulated in PLGA NPs induced higher lachrymal IgG and serum antibody (IgG, IgM and HI) upon subcutaneous delivery. Chitosan and mannan coated PLGA NPs induced higher lachrymal IgG and IgA as well as serum antibody when delivered in a prime-boost strategy by the ocular and nasal routes. In both mucosal and parenteral vaccination approaches, although NPs delivery enhanced Ab-mediated immune responses, at least one booster vaccination was required to generate significantly higher amounts of antibody responses. Previously, it has been reported that nasal vaccination of mice with influenza encapsulated in PLGA NPs without surface modification induced lower IgA in nasal washes due to rapid clearance of the particles from the nasal mucosa [54]. Moreover, mucosal delivery (via aerosol, intranasal and pulmonary routes) of inactivated AIV vaccines combined with conventional and molecular adjuvants failed to induce mucosal IgA in chickens [55–57]. However, a combination of appropriate delivery vehicles, adjuvants and repeated booster vaccinations may influence AIV vaccine efficacy administered through the mucosal routes. A cationic polymer, polyethyleneimine, and a subunit vaccine derived from HA1-2 of H7N9 elicited serum IgG and IgA in the nasal washes of chickens [58]. Our previous work, also showed lachrymal IgA production in chickens upon repeated (at least three times) aerosol delivery of inactivated AIV combined with CpG ODN encapsulated in PLGA NPs [59].

Co-encapsulation of vaccines and adjuvants in our study may ensure physical contacts between antigens and adjuvants in the same NPs. Following phagocytosis by APCs, vaccines and adjuvants can be released simultaneously for efficient antigen presentation and co-stimulation. The released CpG ODN from the NPs may sustain innate immune stimulation [41] for further shaping adaptive immune responses. The current study also demonstrated that CpG ODN inclusion in primary vaccination is critical for inducing better immune responses. This may be explained in such a way that B cells stimulated through Toll-like receptors in the primary vaccination may require minimal adjuvants during the boost to undergo proliferation, differentiation and antibody production. Following intra-nasal administration in mice, CpG ODNs were found to recruit DCs to the nasal epithelial cells forming transepithelial dendrites (TEDs), which may facilitate the capture of inactivated AIV from the nasal mucosal surfaces [60]. In mice, Yin and co-workers also reported the formation of TEDs in the intestine by orally administered CpG ODN and such adjuvant property may have implications for the generation of antigen-specific mucosal immunity [61].

Generally, mucosal vaccination has low efficiency for non-replicating and subunit vaccines compared to parenteral vaccination, because in the case of mucosal vaccination, vaccine uptake is impeded by the various anatomical and physiological barriers present along the mucosal surfaces. Therefore, incorporating cationic polymers [33] and M cells- or APCs-targeting molecules [37,62] to modify the surfaces of PLGA NPs entrapping both AIV and CpG ODN may enhance delivery of the cargo to mucosal APCs. M cells, present within the follicle-associated epithelium that overlies the mucosal-associated lymphoid tissues (the major inductive site of mucosal immunity), sample antigens present on mucosal surfaces and shuttle these antigens to APCs for presentation to CD4+ and CD8+T cells [63,64]. Results of previous studies in mice support our findings in that M cell-targeted PLGA-lipid NPs encapsulating a TLR-ligand and a vaccine antigen boost mucosal immunity [36,37]. In addition to polymeric NPs, mannosylated niosomes encapsulated tetanus toxoid administered orally in rats elicited a significant amount of secretory IgA (sIgA) in mucosal secretions as well as systemic antibody responses [65]. In chickens, there is evidence for the presence of M cells in the follicular epithelium that overlies the gut-and bronchus-associated lymphoid tissues as well as in the bursa of Fabricius [66,67]. The presence of choanal cleft in chickens results in the ingestion of substantial amounts of intranasally administered vaccines and in such scenario, M cells residing in the Peyer’s patches or cecum may be involved in vaccine antigen sampling [67]. Recent study showed induction of mucosal immune responses to recombinant viral vaccine containing M cell targeting motif delivered orally in chickens [68]. In our recent study, we showed that orally administered CpG ODN encapsulated in PLGA NPs induced the expression of higher levels of cytokines and host defense peptide genes in the ileum and cecal tonsils [69]. Although the proportions of M-cells in mucosal tracts (gut and respiratory tracts) of chickens are not known, an approximate 5% of the epithelial cells in the intestinal tracts in humans and 10% in mice constitute M cells [70].

Compared to mannan coated PLGA NPs, chitosan coated PLGA NPs induced higher AIV-specific mucosal antibodies, particularly of IgA isotype. Other studies have demonstrated that chitosan NPs improve the immunogenicity of an intranasally administered DNA vaccine against Newcastle disease virus [71]. The enhanced mucosal immunity resulting from chitosan coated PLGA NPs may be attributed to the residence time of the NPs in the respiratory tract, which prolongs interactions between NPs and mucus for facilitating NPs permeation through the mucosa for better antigen uptake and presentation [32,72]. In contrast, NPs prepared from PLGA alone are negatively charged and lack electrostatic interactions with the negatively charged sialic acid groups of mucin and as such, induce little or no mucosal IgA responses [73]. In mice, AIV encapsulated in a chitosan-poly-(ε-caprolactone) NPs were found to induce higher IgG2a antibodies when administered intranasally, and such responses persisted for a longer time due to a slow-release of antigens from the NPs [74]. Although we did not assess secretory IgA (sIgA) in the intestinal washes, experiments in mice indicated significant amounts of sIgA production in the intestines and respiratory tracts for intranasally administered polyester coated PLGA-NPs [31]. The distant mucosal immunity as well as systemic immunity induced following mucosal application of NPs-based vaccines are believed to be due to trafficking of APCs phagocytizing NPs for presenting antigens to B and T cells residing in other lymphoid tissues [75]. Cationic NPs have been shown to increase mucosal antibody production following pulmonary or intranasal administration mainly due to transfection of resident APCs [74]. Triggering of innate responses involving pro-inflammatory cytokines [35] and type I IFNs [34] by chitosan, may enhance the maturation and activation of professional APCs, which are essential for eliciting adaptive immunity to vaccines. Although the role of para-cellular absorption in vaccine uptake is unclear, cationic NPs in general have been found to open the tight junctions between epithelial cells [76].

Towards developing novel mucosal vaccine and adjuvants, several studies have evaluated particulate vaccine uptake by immune system cells residing on mucosal surfaces and the magnitude and quality of immune responses elicited [77]. To this end, a study in chickens recovered antigen coated polystyrene NP beads from the nasal-associated lymphoid tissues (NALT) and esophagus following intranasal administration [78]. When applied ocularly, most beads were recovered from the inductive mucosal sites including NALT, conjunctival-associated lymphoid tissues (CALT), Harderian glands and trachea [78]. The uptake of lipopolysaccharides or AIV-coated nanobeads by mononuclear phagocytic cells of the respiratory tract in chickens resulted in the upregulation of major histocompatibility complex (MHC) class II and costimulatory molecules, both having relevance in antigen processing and presentation [77]. Another study has also identified an efficient transport of NPs through the follicle-associated epithelium of the nasal cavity and most NPs were found to be deposited in the lymphoid follicles of the NALT and such transport of NPs has been found to be increased by incorporation of CpG ODN or sodium cholate [79]. Even if we did not evaluate particle trafficking, particulate materials may enter the cells through multiple cross-talk pathways (endocytic and autophagy pathways), often recycling of NPs between the early- and late-endosomes and lysosomes for degradation [80] may enhance antigen presentation through MHC class I or II molecules

In AIV infections, protective antibody-mediated immune responses developed on mucosal surfaces following mucosal vaccination may be of paramount importance for inhibiting the binding of the virus to host cell receptors at the site of pathogen entry and thus interrupt the transmission cycle of the virus. However, the systemic immunity induced by parenteral vaccination may not prevent initial virus replication at the port of AIV entry, but may help to counteract virus dissemination [6,7]. Therefore, based on the results presented from the two routes of immunization, most chickens had detectable antibody-mediated responses and this clearly verified immunogenicity of PLGA-NPs-based avian influenza antigens. Although IgG subclasses that enable to assess the quality of Abs are not defined in chickens, combination of PLGA NPs delivery system and mucosal application resulted in a higher magnitude of mucosal immunity. However, in chickens it remains to be tested whether mucosal IgG and IgA contribute to protection against AIV.

In conclusion, the results of the present study demonstrated the effectiveness of PLGA NPs-based and surface modified vaccine formulations in combination with molecular adjuvants for inducing AIV antigen-specific mucosal and systemic antibody responses in chickens. In the future, a more comprehensive study of the impact of this delivery system as a platform for mass vaccination against highly virulent viral respiratory pathogens in a vaccination and challenge model is warranted.
